# 23. Development and Validation of a Risk Score for Post-transplant Lymphoproliferative Disorders among Solid Organ Transplant Recipients

**DOI:** 10.1093/ofid/ofab466.023

**Published:** 2021-12-04

**Authors:** Quenia dos Santos, Neval E Wareham, Amanda Mocroft, Allan Rasmussen, Finn Gustafsson, Michael Perch, Søren Schwartz Sørensen, Oriol Manuel, Nicolas Mueller, Jens Lundgren, Joanne Reekie

**Affiliations:** 1 Centre of Excellence for Health, Immunity and Infections (CHIP), Rigshospitalet, University of Copenhagen, Copenhagen, Copenhagen, Hovedstaden, Denmark; 2 Department of Abdominal Surgery, Rigshospitalet, University of Copenhagen, Copenhagen, Denmark, Copenhagen, Hovedstaden, Denmark; 3 Department of Cardiology, University of Copenhagen, Copenhagen, Denmark, Copenhagen, Hovedstaden, Denmark; 4 Department of Cardiology, Section for Lung Transplantation, Rigshospitalet, University of Copenhagen, Copenhagen, Hovedstaden, Denmark; 5 Department of Nephrology, Rigshospitalet, University of Copenhagen, Copenhagen, Denmark, Copenhagen, Hovedstaden, Denmark; 6 Lausanne University Hospital and University of Lausanne, Lausanne, Luzern, Switzerland; 7 University Hospital Zurich, Zürich, Switzerland, Swiss Transplant Cohort Study, Zurich, Zurich, Switzerland; 8 Copenhagen University Hospital, Rigshospitalet, Copenhagen, Denmark, Copenhagen, Hovedstaden, Denmark; 9 Centre of Excellence for Health, Immunity and Infections (CHIP) & PERSIMUNE, Copenhagen University Hospital, Rigshospitalet, Copenhagen, Denmark, Copenhagen, Hovedstaden, Denmark

## Abstract

**Background:**

Post-transplant lymphoproliferative disease (PTLD) is a well-recognized complication after transplant. This study aimed to develop and independently validate a risk score to predict PTLD among solid organ transplant (SOT) recipients (kidney, liver, lung and heart).

**Methods:**

Poisson regression identified predictors of PTLD with the best fitting model selected for the risk score, where each predictor contributed with a risk coefficient to the risk score, dividing patients in high vs low risk of having a PTLD.

**Results:**

For both cohorts, most of the patients were male, aged more than 16 years old, kidney recipients and with a low-risk pre-transplant Epstein-Barr Virus (EBV) IgG donor/recipient serostatus. The derivation cohort consisted of 2546 SOT transplanted at Rigshospitalet, Copenhagen between 2004-2019; 57 developed PTLD. Predictors of PTLD were high-risk pre-transplant Epstein-Barr Virus (EBV) IgG donor/recipient serostatus, and current plasma EBV DNA positive, abnormal hemoglobin and C-reactive protein levels. A positive EBV DNA was the strongest parameter for the PTLD risk score (figure 1), although the model was able to predict the risk of PTLD cases in both EBV positive and EBV negative individuals. Individuals in the high-risk group had almost 7 times higher incidence of PTLD compared to the low risk group (table 1). In the validation cohort of 1611 SOT recipients between 2008-2018 from University Hospital of Zürich, 24 developed PTLD. A similar seven times higher risk of PTLD was observed in the high-risk group compared to the low risk group (table 1). The discriminatory ability was also similar in derivation (Harrell’s C-statistic of 0.82 95%CI (0.76-0.88) and validation (0.82, 95% CI:0.72-0.92) cohorts.

An explanation about how the risk for PTLD is calculated for the SOT recipients; in this example the risk of developing PTLD is calculated in the next 180 days

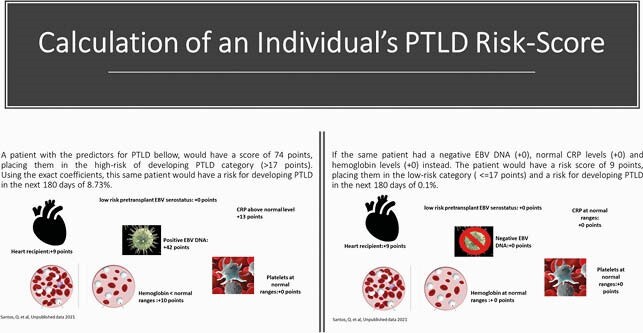

Performance of the PTLD score in the derivation and validation cohorts (low-risk group: score<=17 points; high-risk group: score>17 points)

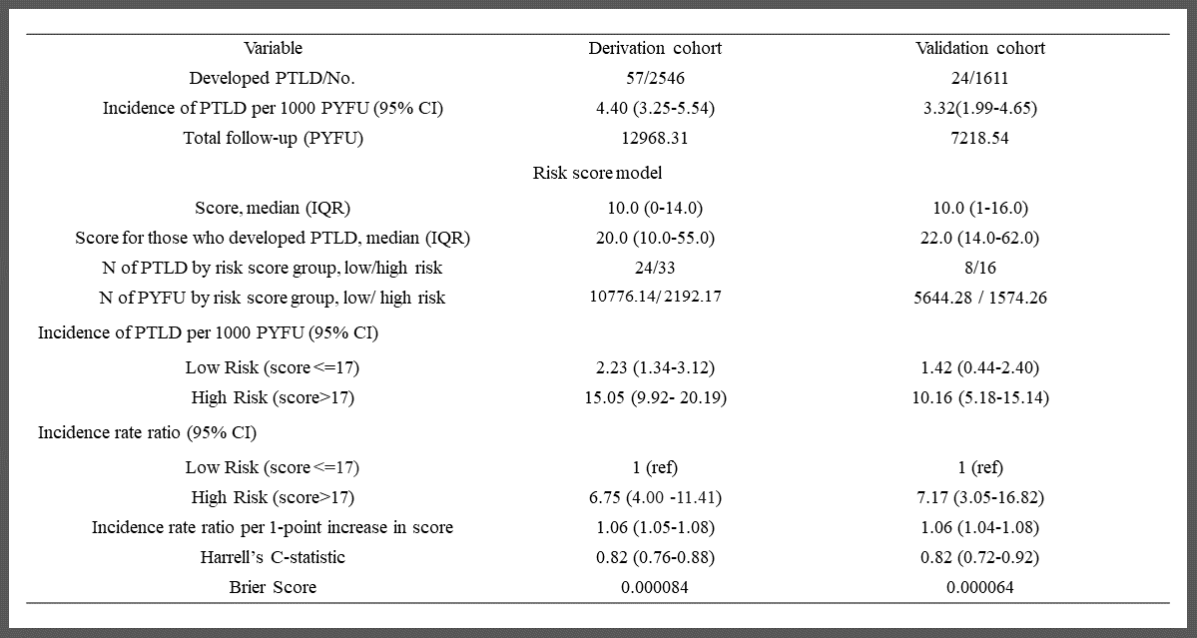

**Conclusion:**

The risk score had a good discriminatory ability in both cohorts and helped to identify patients with higher risk of developing PTLD, so they can be monitored more often. This is the first risk-score developed and externally validated to predict risk of PTLD among SOT recipients.

**Disclosures:**

**All Authors**: No reported disclosures

